# Supplementary material for: Functional Copy-Number Alterations as Diagnostic and Prognostic Biomarkers in Neuroendocrine Tumors
Source: Int J Mol Sci. 2024 Jul 9;25(14):7532. doi: 10.3390/ijms25147532 (PMC11277019; doi:10.3390/ijms25147532)
Supplement: Supplementary file 1 [file ijms-25-07532-s001.zip › Supplementary Figures.pptx]

## Slide 1
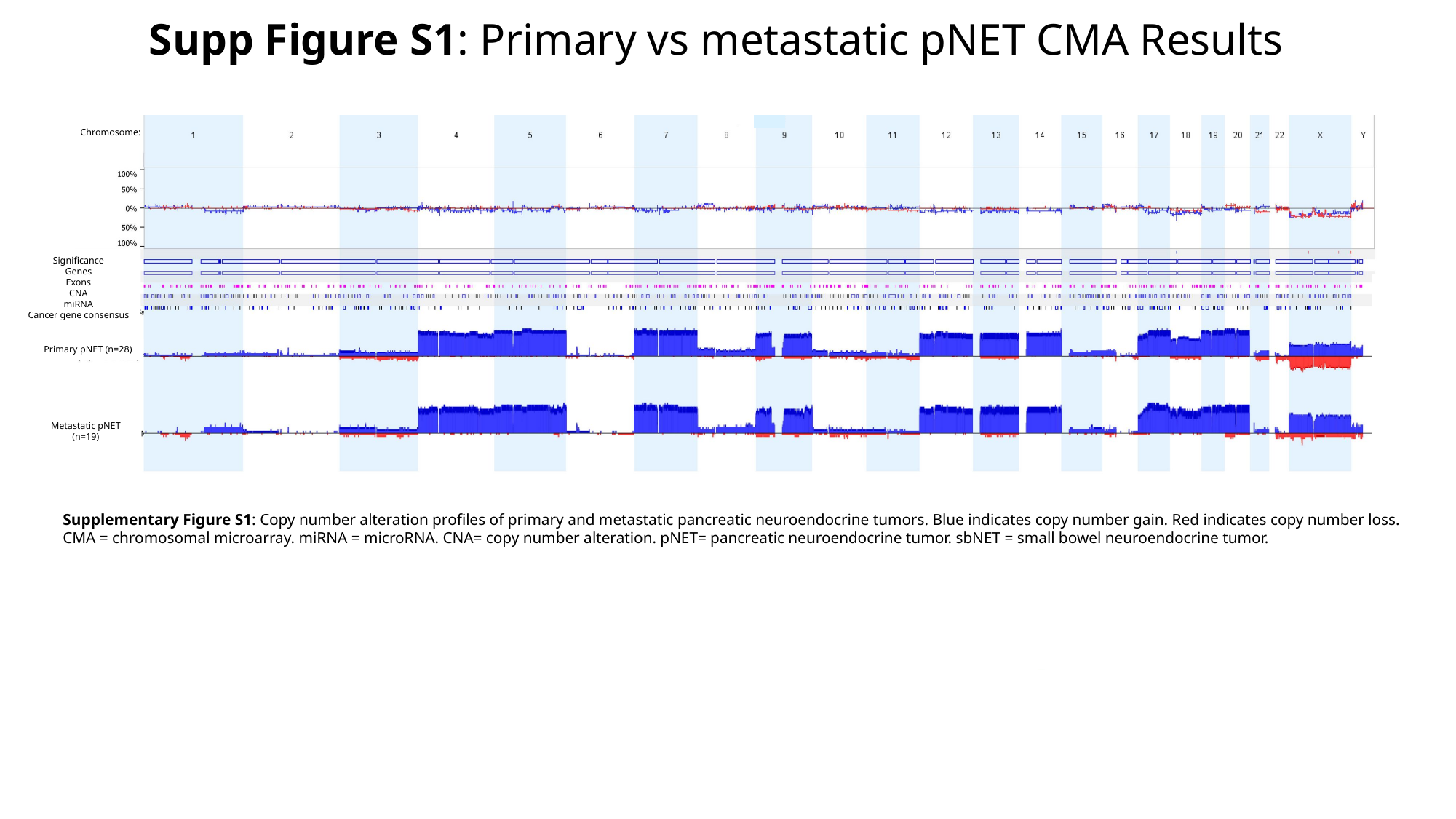

Supp Figure S1: Primary vs metastatic pNET CMA Results
Chromosome:
Significance
Genes
Exons
CNA
miRNA
Cancer gene consensus
Primary pNET (n=28)
Metastatic pNET (n=19)
Supplementary Figure S1: Copy number alteration profiles of primary and metastatic pancreatic neuroendocrine tumors. Blue indicates copy number gain. Red indicates copy number loss.
CMA = chromosomal microarray. miRNA = microRNA. CNA= copy number alteration. pNET= pancreatic neuroendocrine tumor. sbNET = small bowel neuroendocrine tumor.

## Slide 2
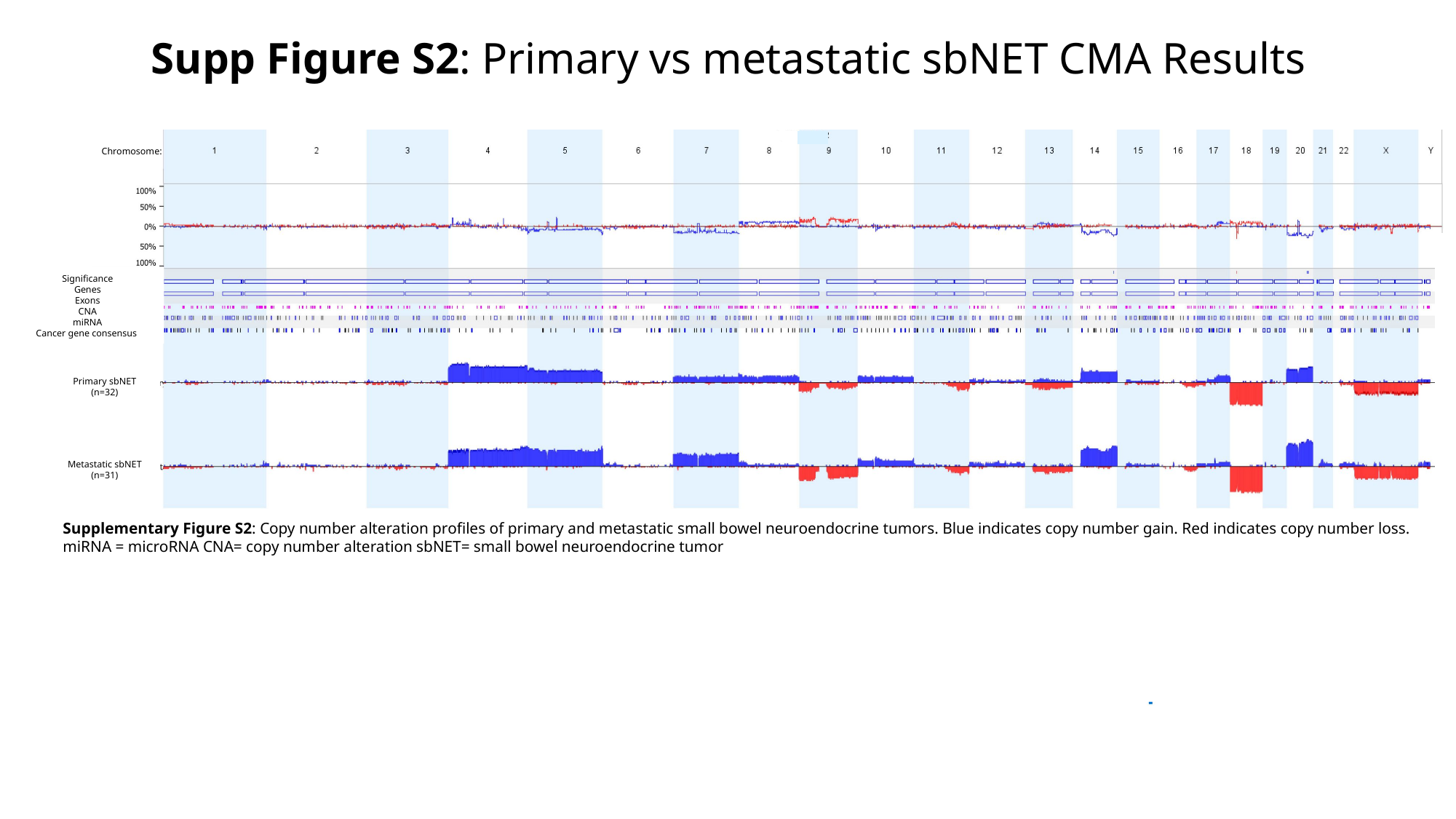

Supp Figure S2: Primary vs metastatic sbNET CMA Results
Chromosome:
Significance
Genes
Exons
CNA
miRNA
Cancer gene consensus
Primary sbNET (n=32)
Metastatic sbNET (n=31)
Supplementary Figure S2: Copy number alteration profiles of primary and metastatic small bowel neuroendocrine tumors. Blue indicates copy number gain. Red indicates copy number loss.
miRNA = microRNA CNA= copy number alteration sbNET= small bowel neuroendocrine tumor

## Slide 3
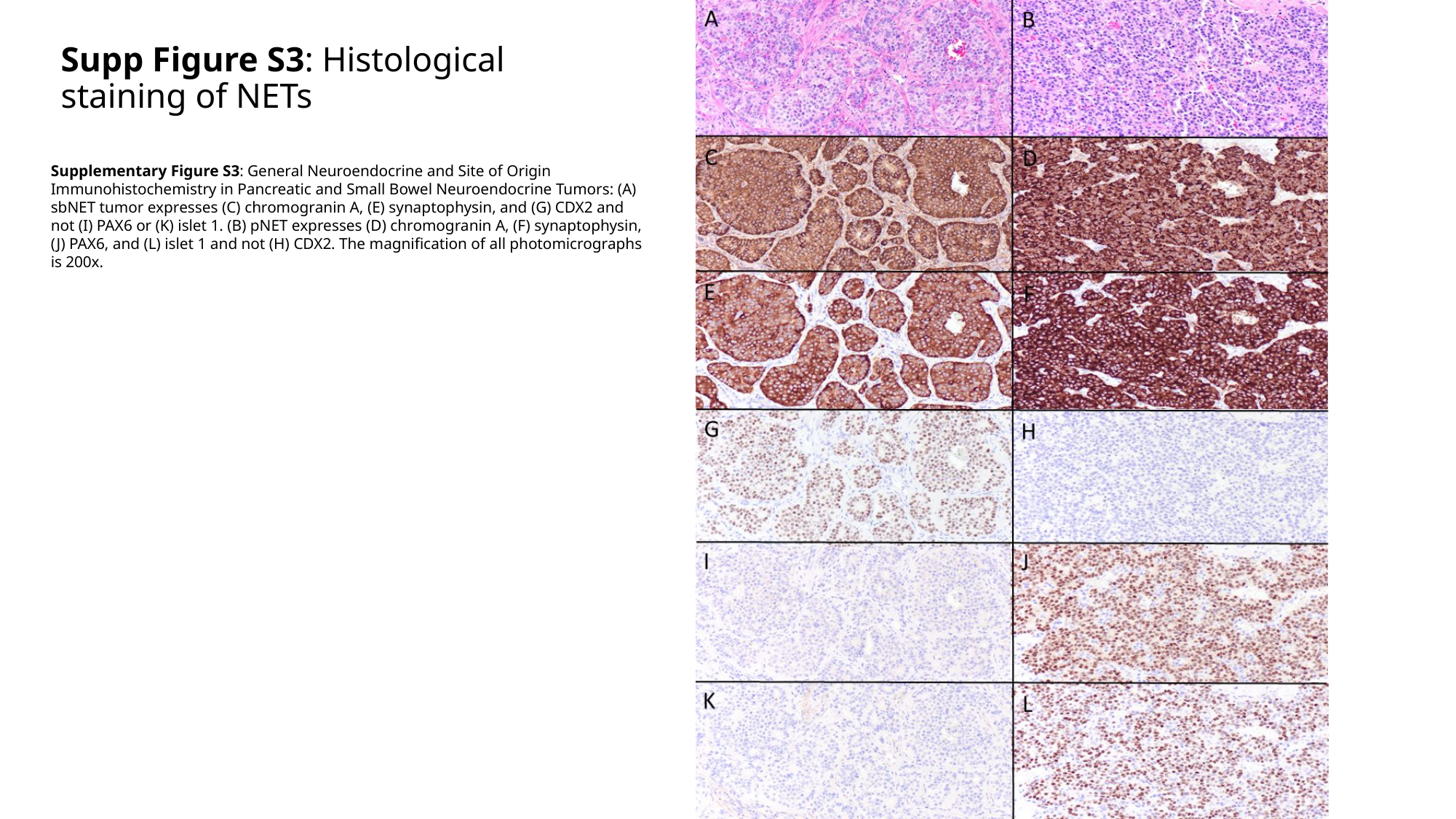

Supp Figure S3: Histological staining of NETs
Supplementary Figure S3: General Neuroendocrine and Site of Origin Immunohistochemistry in Pancreatic and Small Bowel Neuroendocrine Tumors: (A) sbNET tumor expresses (C) chromogranin A, (E) synaptophysin, and (G) CDX2 and not (I) PAX6 or (K) islet 1. (B) pNET expresses (D) chromogranin A, (F) synaptophysin, (J) PAX6, and (L) islet 1 and not (H) CDX2. The magnification of all photomicrographs is 200x.
